# Measuring the Usability and Quality of Explanations of a Machine Learning Web-Based Tool for Oral Tongue Cancer Prognostication

**DOI:** 10.3390/ijerph19148366

**Published:** 2022-07-08

**Authors:** Rasheed Omobolaji Alabi, Alhadi Almangush, Mohammed Elmusrati, Ilmo Leivo, Antti Mäkitie

**Affiliations:** 1 Research Program in Systems Oncology, Faculty of Medicine, University of Helsinki, 00100 Helsinki, Finland; alhadi.almangush@helsinki.fi (A.A.); antti.makitie@helsinki.fi (A.M.); 2 Department of Industrial Digitalization, School of Technology and Innovations, University of Vaasa, 65200 Vaasa, Finland; moel@uwasa.fi; 3 Department of Pathology, University of Helsinki, Haartmaninkatu 3 (P.O. Box 21), FIN-00014 Helsinki, Finland; 4 Institute of Biomedicine, University of Turku, Pathology, 20500 Turku, Finland; ilmo.leivo@utu.fi; 5 Faculty of Dentistry, Misurata University, Misurata 2478, Libya; 6 Department of Otorhinolaryngology—Head and Neck Surgery, University of Helsinki, Helsinki University Hospital, 00029 HUS Helsinki, Finland; 7 Department of Clinical Sciences, Intervention and Technology, Division of Ear, Nose and Throat Diseases, Karolinska Institute, Karolinska University Hospital, 17177 Stockholm, Sweden

**Keywords:** machine learning, explainability, usability, prognostication, web-based tool

## Abstract

**Background**: Machine learning models have been reported to assist in the proper management of cancer through accurate prognostication. Integrating such models as a web-based prognostic tool or calculator may help to improve cancer care and assist clinicians in making oral cancer management-related decisions. However, none of these models have been recommended in daily practices of oral cancer due to concerns related to machine learning methodologies and clinical implementation challenges. An instance of the concerns inherent to the science of machine learning is explainability. **Objectives:** This study measures the usability and explainability of a machine learning-based web prognostic tool that was designed for prediction of oral tongue cancer. We used the System Usability Scale (SUS) and System Causability Scale (SCS) to evaluate the explainability of the prognostic tool. In addition, we propose a framework for the evaluation of post hoc explainability of web-based prognostic tools. **Methods:** A SUS- and SCS-based questionnaire was administered amongst pathologists, radiologists, cancer and machine learning researchers and surgeons (*n* = 11) to evaluate the quality of explanations offered by the machine learning-based web prognostic tool to address the concern of explainability and usability of these models for cancer management. The examined web-based tool was developed by our group and is freely available online. **Results:** In terms of the usability of the web-based tool using the SUS, 81.9% (45.5% strongly agreed; 36.4% agreed) agreed that neither the support of a technical assistant nor a need to learn many things were required to use the web-based tool. Furthermore, 81.8% agreed that the evaluated web-based tool was not cumbersome to use (usability). The average score for the SCS (explainability) was 0.74. A total of 91.0% of the participants strongly agreed that the web-based tool can assist in clinical decision-making. These scores indicated that the examined web-based tool offers a significant level of usability and explanations about the outcome of interest. **Conclusions:** Integrating the trained and internally and externally validated model as a web-based tool or calculator is poised to offer an effective and easy approach towards the usage and acceptance of these models in the future daily practice. This approach has received significant attention in recent years. Thus, it is important that the usability and explainability of these models are measured to achieve such touted benefits. A usable and well-explained web-based tool further brings the use of these web-based tools closer to everyday clinical practices. Thus, the concept of more personalized and precision oncology can be achieved.

## 1. Introduction

A momentous drive to apply artificial intelligence or its subfield, machine learning in diagnostic medicine has emerged in recent times. This is because recently developed machine learning models have been reported to improve cancer management by enhancing prognostication and offering precision medicine [1,2]. The most recent evolution of machine learning, that is, deep learning, has shown the ability to handle extremely large datasets such as image and gene expression data [3,4,5]. Additionally, it has a more complex functionality that can assist the deep learning models to make a more accurate prognostication [5].

Despite these touted benefits of the application of machine learning in medical diagnostics [6,7,8,9], certain concerns have limited the application of machine learning models in daily clinical practices [10]. These include ethical concerns, understanding the science of machine learning techniques, and the clinical implementation of these models [10,11]. Ethical concerns addressed data privacy and confidentiality, peer disagreement (contradictory diagnostic or prognostic opinion between the model and the clinician), patient’s liberty to impact the chosen treatment, and potential changes in the patient–clinician relationship to a three-way paradigm (patients–machine learning models–clinicians) [10]. An example of the concerns that are inherent to the science of machine learning is the lack of transparency (black-box approach) [11,12]. That is, the models cannot explicitly provide a required explanatory rationale behind the result (i.e., decision) generated [13].

Understanding the rationale behind the predictions of these models is crucial to foster a reasonable level of trust in the results generated by these models. One of the approaches to achieve some level of trust in the developed models is to improve the quality of explanations offered by these models [14]. Several attempts have been made to improve the efficiency and effectiveness of the explanations given by the machine learning models [15,16]. For example, recent studies have proposed the local interpretable model-agnostic explanations (LIME) and Shapley additive explanations (SHAP) frameworks for model explainability [17,18,19]. However, most of the efforts that involve the use of LIME and SHAP are useful to provide interpretations and explanations before the integration of these models as a web-based tool. Hence, attention should also be given to the explainability and usability of machine learning models that have been integrated as a web-based tool. Having an explainable and usable web-based tool is poised to bring its adoption closer to reality in daily clinical practices [11]. Recently, few studies have evaluated utility features, such as simplicity and response time of a machine learning model in an application [13,19,20]. Although not much has been done regarding the causability of web-based tools. Therefore, we aim to suggest the use of system usability scale (SUS) and system causability scale (SCS) to measure the quality of explanations and usability of a web-based tool.

In this study, we seek to measure the quality of explanations offered by a machine learning-based web prognostic tool for prognostication in oral tongue cancer. The quality of the explanations is measured from the medical users’ (oncologists and cancer researchers) perspectives. This is important due to the quest for personalized medicine. Despite the reported performance of machine learning models in prognostication of oral cancer, none of these models have been integrated as a web-based tool for this purpose. Integrating the trained and internally and externally validated model as a web-based tool or calculator is poised to help in the quest for patient-centered oncological care and precision medicine [21]. To ensure that the afore-mentioned benefits are achieved, it is important that the quality of explanations offered by these tools are measured for accuracy.

## 2. Material and Methods

### 2.1. A Web-Based Prognostic Tool

The integration of machine-learning models as a web-based tool has gained momentum in recent years. The motivation for this integration is to facilitate the adoption of these models in clinical practices [21]. For any web-based tool to be recommended for use in daily clinical practices, it is important for such a tool to demonstrate high-level of usability with unique and high-quality explanations. We demonstrated a novel approach to measuring the quality of explanations and usability of a machine-learning web-based tool using system usability scale and system causability scale on a machine-learning web-based tool developed by our group [1]. The tool was developed using an artificial neural network to predict locoregional recurrences in early-stage oral tongue cancer [1]. The tool was developed using cohorts from five (5) Finnish University Hospitals and externally validated with a cohort from a cancer center in Brazil. Considering the aim of the study in measuring the quality of explanations and usability of an already developed web-based tool that is freely available, the details of the training process of the model is beyond the scope of the objectives of this study as it has already been published [1]. The tool is freely available at http://oncotelligence.com/ (accessed on 19 May 2022) (Prediction of risk of locoregional recurrences in early oral tongue cancer (Oral Tongue Squamous Cell Carcinoma (OTSCC))).

### 2.2. Questionnaire

#### 2.2.1. Design and Participants

The participants were randomly selected from pathologists, radiologists, surgeons, cancer researchers and machine-learning researchers who have recently published a study relating to the application of machine learning or deep learning in the prognostication of outcome in either oral cancer or head and neck cancer in the last five years (2017–2021). This set of participants were identified as the potential users of a web-based prognostic tool. Therefore, a total of 16 potential participants were identified and recruited (i.e., 5 participants each from cancer and machine-learning researchers and 2 participants each from pathologists, radiologists, and surgeons).

A relatively small number of participants were invited to answer to the SUS- and SCS-based questionnaire. Participants were guaranteed anonymity to ensure freedom of participation and response. The duration to participate in the questionnaire was set to one month with reminders sent each week. All procedures performed in this study were in accordance with the World Medical Association Declaration of Helsinki and its later amendments on comparable ethical standards. Participation in the questionnaire was voluntary, thus, the responses were processed and analyzed anonymously.

#### 2.2.2. Questionnaire Development

The System Usability Scale (SUS) offers a valid, quick, easy but reliable way of measuring the usability of a system [22]. Therefore, this tool was selected because it has become an industrial mainstay for usability evaluation. Basically, it consists of a 10-item questionnaire with five response options for the participants (Supplementary Material). These options range from ‘strongly agree’ to ‘strongly disagree’ (Table 1). To evaluate the web-based tool, we modified these 10-item questions to fit the objectives of this study while retaining the themes of each question (Supplementary Material). The System Causability Scale (SCS), on the other hand, was based on the study by Holzinger et al., which proposed this scale [12] (Table 2). However, in this study, we expanded the use of this tool into the medical domain, specifically for outcome prognostication in tongue cancer. In addition, we administered it among various experts in this domain.

#### 2.2.3. Questionnaire Validation

Even though both SUS and SCS are well-known tools, two independent reviewers (R.A and A.A) accessed the questionnaire reliability and sensitivity to address the stated objectives of this study. Additionally, another independent reviewer (A.M) examined the questionnaire validity. There were consensus meetings and discussions to resolve possible discrepancies in the questions or theme contained in the questionnaire before it was sent out to potential participants. The final questionnaire with high inter-observer reliability among these three independent reviewers was sent to respondents (Supplementary Material).

### 2.3. Measuring the Quality of Explanations

The quality of explanations offered by our web-based prognostic tool is measured by the SUS [23] (Table 1). The main theme of the SUS is to measure the simplicity of the usage of the web-based tool, and most importantly, the usability of the web-based tool. Considering the concerns of using SUS in terms of the interpretation of results and the assumption that it is unidimensional [12], factors relating to usability (item number 8) and learnability (items number 4 and 10) were used in this study (Table 1). Additionally, the explainability of the model (understanding the model, its parameters and how it works) is measured using the SCS [12] (Table 2). This methodology was based on the studies by Holzinger et al., who proposed this scale [12].

## 3. Results

### 3.1. Participants Description

Eleven (68.8%) out of the 16 recruited participants answered the questionnaire and their responses were analyzed. This cohort included five (45.5%) cancer-researchers, three (27.3%) machine-learning researchers, and one (9.1%) participant each from pathology, radiology, and surgery. The study group comprised ten males and one female and their age range was typically between 30–50 years. All the participants had a minimum of 2 years of working experience in oncology (or related field) as a pathologist, radiologist, surgeon, cancer-related researcher, or machine-learning researcher. A significant proportion (45.5%) of the participants had over 10 years of experience.

### 3.2. Usability and Explainability

**Figure 1:**
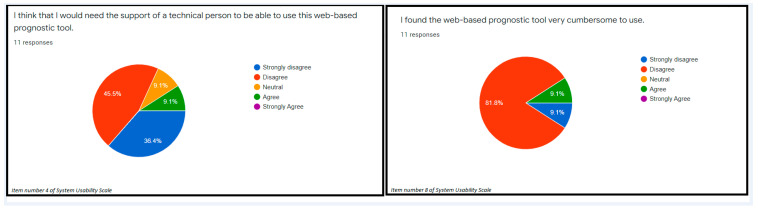
The usability of a web-based prognostic tool using items number 4 and 8 of the System Usability Scale.

**Figure 2:**
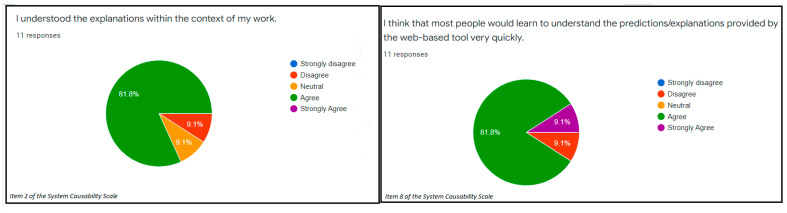
The explainability of a web-based prognostic tool using items number 2 and 8 of the System Causability Scale.

**Figure 3:**
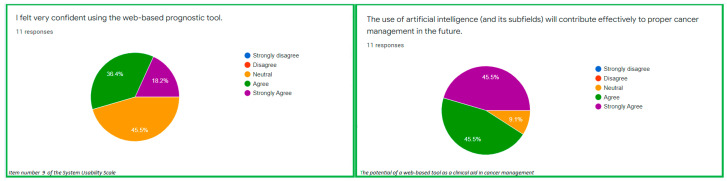
The potential of a web-based tool in cancer management.

The items number 4 and 10 of the System Usability Scale measured the usability of the evaluated web-based prognostic tool. In terms of the usability, 45.5% strongly disagreed while 36.4% disagreed that the proper use of the web-based prognostic tool warranted either a lot of learning or support of a technical support person (Figure 1). Considering the usability in terms of how cumbersome it is to use the web-based prognostic tool, 81.8% disagreed that the web-based tool was complex to use (usability) (Figure 1). The average score for the SCS (explainability) was 0.74. In addition, the explainability of the web-based tool based on the SCS was presented in Figure 2. A total of 91.0% of the participants strongly agreed that the web-based tool is poised to provide a second opinion to the clinicians during clinical decision-making (Figure 3). These results indicated that the examined web-based tool offers a significant amount of usability and explanations about the outcome of interest. However, the SUS revealed that the usability confidence (item 9) should be improved as 36.4% had only little confidence using the web-based tool (Figure 3).

### 3.3. Fidelity and Interpretability

**Figure 4:**
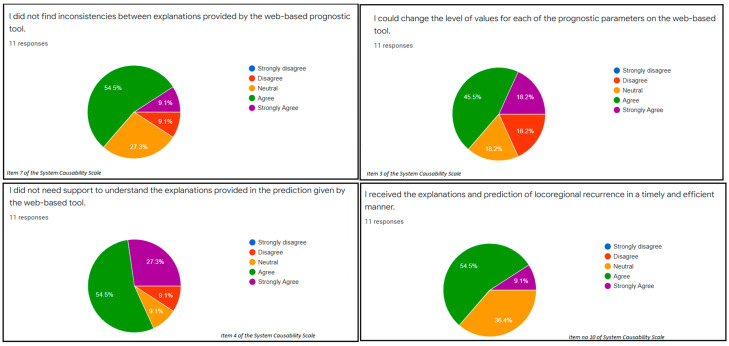
Measuring the essential components of explainability using the System Usability Scale. The fidelity includes soundness and completeness (items 7 and 3 of SUS), while interpretability includes parsimony and clarity (items 4 and 10 of SUS).

For fidelity components, 9.1% strongly agreed while 54.5% agreed about the soundness of the model (Figure 4: item 7 of the SCS). Similarly, 18.2% strongly agreed while 45.4% agreed about the completeness of the web-based prognostic tool (Figure 4: item 3 of the SCS). For the interpretability components, 27.3% strongly agreed about parsimony of the web-based prognostic tool while 54.5% agreed about the parsimony of the tool (Figure 4: item 4 of the SCS). Additionally, 9.1% strongly agreed while 54.5% agreed on the clarity of the examined web-based prognostic tool (Figure 4: item 10 of the SCS).

### 3.4. Framework for an Explainable Web-Based Tool

**Figure 5:**
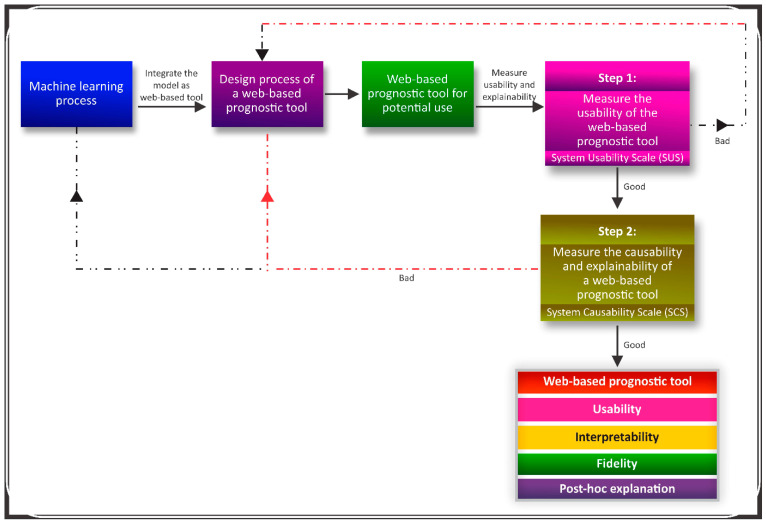
Framework for post hoc explainability of a web-based prognostic tool.

A framework that considers the post hoc explanations of a web-based prognostic tool is presented in Figure 5. The framework considers all the essential components of post hoc explainability. The framework emphasized that the essential components (items 4 and 10) of the SUS should be properly considered after the developed machine-learning model is integrated as a web-based prognostic tool. Similarly, the score of the SCS ranged between 0–1. Therefore, less than 0.5 indicates a need to significantly modify the design of the web-based tool (Figure 1).

## 4. Discussion

**Figure 6:**
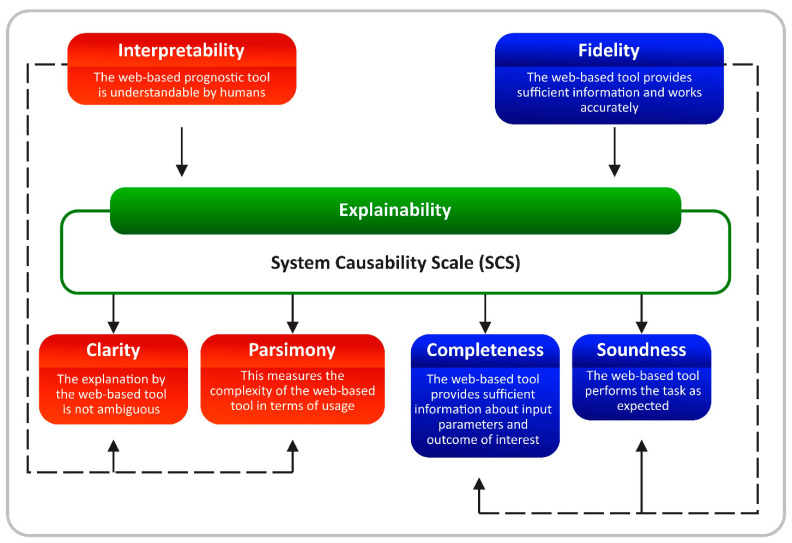
The essential components of explainability.

In this study, we explored the usability and explainability of a machine-learning web-based prognostic tool for oral tongue cancer prognostication. The usability and quality of the explanations were measured by the System Usability Scale (SUS) and System Causability Scale (SCS), respectively. It is important that the usability and explainability of these models are measured to further bring the usage of these web-based tools closer to reality in daily clinical practices. However, there are no standardized methods to measure the quality of the explanations at the moment. Nevertheless, exploring the quality of usability and explainability using SUS and SCS could be a promising approach. While we acknowledge the fact that both explainability and interpretability are sometimes used interchangeably in the literature [24,25], the quality of explanations as presented in this study precisely captured the core components of explainability–interpretability and fidelity [14,26,27] (Figure 6). Following the ill-definition of explainability in most of the published studies [28], it is hoped that both interpretability and fidelity can assist in achieving a reasonable explainability of the web-based tool [29].

The core components of interpretability include clarity and parsimony while the essential components of fidelity encompass completeness and soundness. For interpretability of the web-based tool, clarity means that the explanations offered by this model are not ambiguous [14,27]. Similarly, the parsimony component of interpretability measures the complexity of the web-based tool in terms of the usage [14,27] (Figure 6). Regarding fidelity, completeness means that the web-based tool provides sufficient information about the inputs and the output. However, soundness as an essential component of fidelity ensures that the web-based tool performs the task as expected [26] (Figure 6). While it has been argued that measuring both interpretability and fidelity at the same time could be challenging [14], the SCS proposed by Holzinger et al. offers some important items that can address some of the essential components of interpretability and fidelity needed to achieve an explainable web-based tool that offers post hoc explanation [12]. Specifically, items number 3, 4, 7 and 10 of the SCS arguably discussed completeness, parsimony, clarity, and soundness of a typical AI-based web tool for cancer prognostication (Table 2).

Despite the fact that explainability of machine-learning models is not well defined [30,31], it is important to mention that the measure of the quality of explanations could be domain-specific, qualitative and subjective. It is important to address these challenges by converting these qualitative explanations into quantitative explanations using user studies [30]. Several studies have attempted to offer quantitative explanations on the explainability of these models [32,33]. The use of SCS proposed by Holzinger et al. was able to convert qualitative explanations into a quantitative explanation to offer insightful information regarding the causability of a typical web-based tool. This study corroborates the earlier approach by further examining the usability and causability of these models so as to further ensure a trustworthy model. The use of SCS may ensure that the web-based prognostic tool offers post hoc explanations that provide insights about the inputs and outcome of interest without necessarily knowing the detailed mechanism of action of the tool. This is aimed at ensuring that these assistive tools are understandable by the stakeholders [24,34].

Based on these scales, it was revealed that the usability confidence of the evaluated web-based tool in this study can be improved. An insightful approach to improve the usability confidence is to include some footnotes on the user interface of the web-based tool. This can include some explanations regarding the meaning of the input parameters especially for those who may not have enough technical knowledge. As the web-based tools are targeted to be used by experts in a related field, this may not call for a serious concern. Likewise, another approach that can improve the usability confidence is to include the limitations of this web-based tool as a footnote. This is poised to enhance usability confidence. Furthermore, it was revealed that the prognostic factors or parameters to be used to develop the web-based tool should be related to the targeted outcome. These relationships should be evidence-based, for example, in medical oncology or through published studies. This gives some level of usability confidence to the users of the web-based tool in terms of causability.

The proposed framework in this study offers an opportunity for the early evaluation of the usability and explainability of a potential web-based prognostic tool (Figure 5). As shown in Figure 1, if the usability components of the SUS are low, an interventional approach can be initiated to redesign the web-based prognostic tool to offer improved usability. Likewise, a moderately low score (0.5–0.7) of the SCS of the web-based prognostic tool is an indicator that either the machine-learning process should be revisited to include important parameters in the training, or the web-based tool should be redesigned so that essential details are included to understand the parameters included in the web-based tool (Figure 5). In case of extremely low SCS score (<0.5), both steps of retraining the model to include significant prognostic parameters and rebuilding of the web-based prognostic tool may become inevitable, in fact, it is strongly recommended. Therefore, our proposed framework ensures that a potential web-based prognostic tool is properly scrutinized for potential usage in the medical domain prior to presenting such a tool to the medical community.

The goals of explainability involve trustworthiness, informativeness, fairness, confidence (performance measures), accessibility, interpretability, understandability, causability, transferability, interactivity, and privacy-orientation [30]. It is important to consider these goals during the development of the models and the actual usage of these models as an assistive tool (web-prognostic tool or otherwise). The approach presented in this study is posited to address causability, understandability (predicted results), confidence, interactivity, accessibility, interpretability, and trustworthiness of a potential assistive tool for the management of cancer.

It is of note that the usage of a machine learning-based prognostic tool involving the tool should be trustworthy as clinicians are obliged to make decisions that uphold the basic medical principle of medical ethics (autonomy, beneficence, nonmaleficence and justice) [10,35]. This principle of medical ethics is corroborated by the High-Level Expert Group definition of trustworthy artificial intelligence models [36]. Efforts are made continuously to address the concept of trustworthy artificial intelligence (transparency, credibility, auditability, reliability, recoverability, safety, and ethics) [10,37]. A noticeable step towards achieving a trustworthy AI is to develop an explainable AI [14]. This premise was also supported as the European Institute of Innovation and Technology Health emphasized that explainable, causal, and ethical are important parameters that can facilitate the adoption of artificial intelligence models (systems) in daily clinical practices [38]. Whilst the strategies to develop and regulate trustworthy artificial intelligence models are still evolving, measuring the quality of explanations offered by these models remains pivotal.

Therefore, evaluating the quality of explanations by the duo of SUS and SCS can bring some level of trustworthiness to the web-based prognostic tool or assistive prognostic tools. Using these scales will ensure that the medical experts can easily use this assistive tool. Additionally, they understand the result produced by these web-based tools (interpretability). Several attempts have been made to address the explainability technically (pre hoc explainability) [17,19,30]. However, a few attempts have been published to address the quality of explanations from the end-users’ perspective (post hoc explainability) [30]. One of these is the study by Holzinger et al. that evaluated the quality of explanations offered by the Framingham Risk Tool [12]. In the present study, we addressed the limitations of the study by Holzinger et al. by exploring the quality of explanations of an oral tongue cancer prognostic tool (i.e., in the medical domain).

An assistive tool that offers high quality explanations satisfies completeness, soundness, and some level of interpretability of the model [14]. However, it is important to emphasize that the quest for explainability should not affect the performance metrics (such as accuracy) of the model. Hence, there should be no trade-off between accuracy and explainability [14]. This is why efforts aimed at post hoc explanations as shown in this study become pertinent. Our study has several limitations. One of the limitations is that most of these terms (explainability, transparency, fidelity, usability and causability) are not well-defined in the literature [28]. Therefore, it was challenging to achieve a consensus on these definitions. Public readers, scientific communities and AI enthusiasts may give these terms varying meanings. The need for formal definition of these terms becomes imperative in the future. In addition, the number of respondents to the SUS and SCS-based questionnaire in this study was relatively small. Despite this, the importance of having a usable and explainable web-based tool is necessary, as shown in this study. Likewise, the proposed tools (SUS and SCS) may not address the explainability concerns that are inherent to the process of machine learning. Nevertheless, these tools are essential suggestions to ensure that no further additional explainability and usability issues are added to these models when they are integrated as a web-based tool for further use. The SCS is based on Likert scales which can easily be misinterpreted [39].

The main limitation of this study was the limited number of participants that answered the questionnaire. As the main objective of the study centered on measuring the quality of explanations provided by experts or potential users in the cancer management domain, it would be more desirable to have a relatively balanced representations in terms of their gender, age and other aspects. In addition, the number of participants in each domain was not equally distributed. Considering the objective of this study and limited size of the cohort, we did not include statistical analysis in our results.

## 5. Conclusions

In conclusion, we proposed measuring the usability and explainability of a web-based prognostic tool using the combination of SUS and SCS. This will ensure that the web-based tool has the needed characteristics of interpretability (clarity and parsimony) and fidelity (completeness and soundness). The SUS measures the usability and interpretability of a web-based tool. Conversely, the SCS measures the quality of explanations by the web-based tool. In addition, it ensures that the cause–effect (causality) parameters are included in the web-based tool. The framework proposed in this study is poised to ensure swift intervention either during the development of the model or during integration is provided to ensure that the web-based tool provides the necessary usability and explanations on the prediction made. For example, for medical experts, it is important that the web-based tool is usable and explainable and that necessary parameters are included in the development of the model. Most importantly, the clinicians need to understand the predictions made by the model.

Therefore, measuring the quality of explanations can be an interesting lead to contribute to the explainability of machine learning-based assistive tools. Considering the potential of the machine-learning models to aid medical experts, it is important that these experts are able to understand, re-enact and interact with these tools. Hence, successful explainable machine learning-based assistive tools should have effective user interfaces (usability) and evaluate causability. In the future, the legal and privacy aspects of these tools should be considered alongside explainability in order for these tools to fulfill their enormous opportunities in the medical domain.

## Figures and Tables

**Table 1:** Measuring the usability of a web-based tool the System Usability Scale (SUS) [12].

S/N	System Usability Scale (SUS)	Theme from the SUS	Ratings (%)
**1**	I think that I would like to use this web-based prognostic tool in cancer management.	Readiness to use	Strongly agree: 27.3% Agree: 27.3% Neutral: 27.3% Disagree: 9.1% Strongly disagree: 9.1%
**2**	I found the web-based tool unnecessarily complex.	Web-based tool simplicity	Neutral: 18.2% Disagree: 45.5% Strongly disagree: 36.4%
**3**	I thought the web-based tool was easy to use.	Ease of use of the web-based tool	Strongly agree: 27.3% Agree: 63.6% Disagree: 9.1%
**4**	I think that I would need the support of a technical person to be able to use this web-based prognostic tool.	Need teacher/support to use the web-based tool	Agree: 9.1% Neutral: 9.1% Disagree: 45.5% Strongly disagree: 36.4%
**5**	I found the various functions in this web-based prognostic tool to be well integrated.	Understanding the input parameters	Strongly agree: 18.2% Agree: 63.6% Neutral: 9.1% Disagree: 9.1%
**6**	I thought there was too much inconsistency in this web-based prognostic tool.	Clarity of the web-based tool	Neutral: 27.3% Disagree: 54.5% Strongly disagree: 18.2%
**7**	I would imagine that most people would learn to use this web-based prognostic tool very quickly.	No technicality required	Strongly agree: 36.4% Agree: 45.5% Neutral: 9.1% Disagree: 9.1%
**8**	I found the web-based prognostic tool very cumbersome to use.	Less cumbersome tool	Agree: 9.1% Disagree: 81.8% Strongly disagree: 9.1%
**9**	I felt very confident using the web-based prognostic tool.	Usability confidence	Strongly agree: 18.2% Agree: 36.4% Neutral: 45.5%
**10**	I needed to learn a lot of things before I could get going with this web-based prognostic tool.	Easy to use for everyone	Strongly agree: 18.2% Neutral: 45.5% Disagree: 36.4%

**Table 2:** Measuring the causality and quality of explanations using the System Causability Scale (SCS) with the Framingham Model [12].

S/N	System Causability Scale (SCS)	Theme from the SCS	Ratings Rating (Participants, *n* = 11)	Score
**1**	I found that the prognostic parameters included all relevant known causal factors with sufficient precision and detailed information regarding locoregional recurrence of oral cancer	Causality factors in the data	Agree: 4(6) = 24 Neutral: 3(3) = 9 Disagree: 2(2) = 4	37
**2**	I understood the explanations within the context of my work.	Understood the explanations of the web-based tool	Agree: 4(9) = 36 Neutral: 3(1) = 3 Disagree: 2(1) = 2	41
**3**	I could change the level of values for each of the prognostic parameters on the web-based tool.	Change in detail level of income parameters	Strongly agree: 5(2) = 10 Agree: 4(5) = 20 Neutral: 3(2) = 6 Disagree: 2(2) = 4	40
**4**	I did not need support to understand the explanations provided in the prediction given by the web-based tool.	Need teacher/support to use the web-based tool	Strongly agree: 5(3) = 15 Agree: 4(6) = 24 Neutral: 3(1) = 3 Disagree: 2(1) = 2	44
**5**	I found the predictions by the web-based tool helped me to understand causality (cause and effect regarding recurrence of cancer)	Understanding causality based on the inputs and predicted outcome	Strongly agree: 5(1) = 5 Agree: 4(4) = 16 Neutral: 3(4) = 12 Disagree: 2(2) = 4	37
**6**	I was able to use the explanations of the prediction by the web-based tool with my knowledge base.	Usage of the tool with knowledge with knowledge	Strongly agree: 5(2) = 10 Agree: 4(6) = 24 Neutral: 3(3) = 9	43
**7**	I did not find inconsistencies between explanations provided by the web-based prognostic tool.	No inconsistencies from the web-based tool	Strongly agree: 5(1) = 5 Agree: 4(6) = 24 Neutral: 3(3) = 9 Disagree: 2(1) = 2	40
**8**	I think that most people would learn to understand the predictions/explanations provided by the web-based tool very quickly.	Learn to understand	Strongly agree: 5(1) = 5 Agree: 4(9) = 36 Neutral: 3(1) = 3	44
**9**	I did not need more references in the explanations: e.g., medical guidelines, regulations to understand the web-based prognostic tool.	Needs references to use the web-based tool	Strongly agree: 5(2) = 10 Agree: 4(4) = 16 Neutral: 3(2) = 6 Disagree: 2(2) = 4 Strongly disagree: 1(1) = 1	37
**10**	I received the explanations and prediction of locoregional recurrence in a timely and efficient manner.	Timely and efficient prediction of outcome	Strongly agree: 5(1) = 5 Agree: 4(6) = 24 Neutral: 3(4) = 12	41
				$\mathrm{SCS}=\sum_{i}Rating_{i}/550$

Ratings are: 1 = Strongly disagree, 2 = Disagree, 3 = Neutral, 4 = Agree, 5 = Strongly agree.

## Data Availability

The responses of the participants can be made available to the journal, respecting the anonymity of the respondents, if its editors deem it necessary. Excerpts of any of the items in the SUS or SCS can be obtained from the first authors upon reasonable request.

## Supplementary Materials

The following supporting information can be downloaded at: https://www.mdpi.com/article/10.3390/ijerph19148366/s1. Measuring the usability and quality of explanation of a web-based prognostic tool.
