# Supplementary material for: Measuring the Usability and Quality of Explanations of a Machine Learning Web-Based Tool for Oral Tongue Cancer Prognostication
Source: Int J Environ Res Public Health. 2022 Jul 8;19(14):8366. doi: 10.3390/ijerph19148366 (PMC9322510; doi:10.3390/ijerph19148366)
Supplement: Supplementary file 1 [file ijerph-19-08366-s001.zip › ijerph-1756071-supplementary.pdf]

Hello, welcome to take part in this survey.

Usability & Explainability of a machine learning-based cancer prognostic tool.

*Participations and responses are treated anonymously by default.
** Participation means approval to use your response in the ongoing study.
*** The survey closes one month after the date of initial invitation.

*Required

Aim: This survey measures the usability and quality of explanations of a machine learning web-based cancer prognostic tool.

System Usability Scale (SUS) and System Causability Scale (SCS).

To proceed, Let us meet you!

Nice to meet you.

1. Which option best describes you? *

Mark only one oval.

- Oncologist
- Surgeon
- Radiologist
- Pathologist
- Researcher - Cancer
- Researcher - Machine learning
- Other: ____________________

2. Which option best describes your years of experience? *

Mark only one oval.

- Less than 2 years of experience (< 2 years)
- 2 - 5 years of experience
- 6 - 10 years of experience
- Over 10 years of experience (> 10 years)

3. Which age group describes you? *

Mark only one oval.

- 18 - 30 years
- 31 - 40 years
- 41 - 50 years
- 51 - 60 years
- 61 years & above

4. Which answer describes you? *

Mark only one oval.

- Female
- Male
- Other
- Prefer not to say

Here is the link to the web-based tool

http://oncotelligence.com/

It is a machine learning web-based prognostic tool.

You are free to use any value for each of the parameters.

NB:
The worst pattern of invasion (WPOI) refers to the worst manner of infiltration present at the tumor/host interface.

Lymphocytic host response (LHR) is the density of lymphocytes at the tumor interface

Web-based prognostic tool sample image

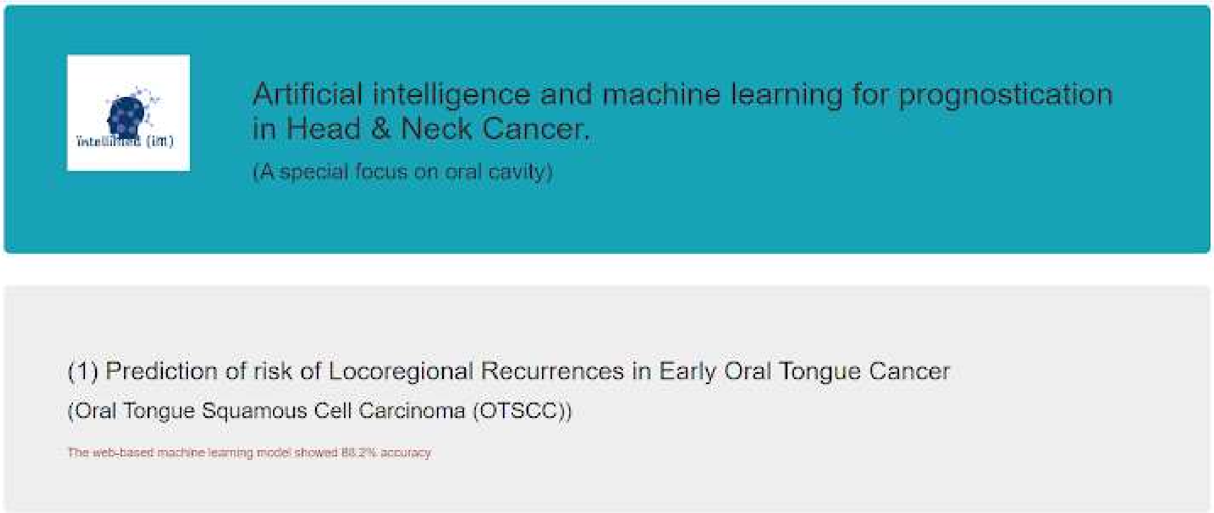

PART I: Measuring the usability of the web-based tool.

System Usability Scale (SUS).

This scale consists of 10 questions.

5. I think that I would like to use this web-based prognostic tool in cancer management. *

Mark only one oval.

- Strongly disagree
- Disagree
- Neutral
- Agree
- Strongly Agree

6. I found the web-based tool unnecessarily complex. *

Mark only one oval.

- Strongly disagree
- Disagree
- Neutral
- Agree
- Strongly Agree

7. I thought the web-based tool was easy to use. *

Mark only one oval.

- Strongly disagree
- Disagree
- Neutral
- Agree
- Strongly Agree

8. I think that I would need the support of a technical person to be able to use this web-based prognostic tool. *

Mark only one oval.

- Strongly disagree
- Disagree
- Neutral
- Agree
- Strongly Agree

9. I found the various functions in this web-based prognostic tool to be well integrated. *

Mark only one oval.

- Strongly disagree
- Disagree
- Neutral
- Agree
- Strongly Agree

10. I thought there was too much inconsistency in this web-based prognostic tool. *

Mark only one oval.

- Strongly disagree
- Disagree
- Neutral
- Agree
- Strongly Agree

11. I would imagine that most people would learn to use this web-based prognostic tool very quickly. *

Mark only one oval.

- Strongly disagree
- Disagree
- Neutral
- Agree
- Strongly Agree

12. I found the web-based prognostic tool very cumbersome to use. *

Mark only one oval.

- Strongly disagree
- Disagree
- Neutral
- Agree
- Strongly Agree

13. I felt very confident using the web-based prognostic tool. *

Mark only one oval.

- Strongly disagree
- Disagree
- Neutral
- Agree
- Strongly Agree

14. I needed to learn a lot of things before I could get going with this web-based prognostic tool. *

Mark only one oval.

- Strongly disagree
- Disagree
- Neutral
- Agree
- Strongly Agree

PART II: Measuring the quality of explanations provided by the web-based tool.

System Causability Scale (SCS).

This scale consists of 10 questions.

15. I found that the prognostic parameters included all relevant known causal factors with sufficient precision and detailed informtion regarding locoregional recurrence of oral cancer. *

Mark only one oval.

- Strongly disagree
- Disagree
- Neutral
- Agree
- Strongly Agree

16. I understood the explanations within the context of my work. *

Mark only one oval.

- Strongly disagree
- Disagree
- Neutral
- Agree
- Strongly Agree

17. I could change the level of values for each of the prognostic parameters on the web-based tool. *

Mark only one oval.

- Strongly disagree
- Disagree
- Neutral
- Agree
- Strongly Agree

18. I did not need support to understand the explanations provided in the prediction given by the web-based tool. *

Mark only one oval.

- Strongly disagree
- Disagree
- Neutral
- Agree
- Strongly Agree

19. I found the predictions by the web-based tool helped me to understand causality (cause and effect regarding recurrence of cancer). *

Mark only one oval.

- Strongly disagree
- Disagree
- Neutral
- Agree
- Strongly Agree

20. I was able to use the explanations of the prediction by the web-based tool with my knowledge base. *

Mark only one oval.

- Strongly disagree
- Disagree
- Neutral
- Agree
- Strongly Agree

21. I did not find inconsistencies between explanations provided by the web-based prognostic tool. *

Mark only one oval.

- Strongly disagree
- Disagree
- Neutral
- Agree
- Strongly Agree

22. I think that most people would learn to understand the predictions/explanations provided by the web-based tool very quickly. *

Mark only one oval.

- Strongly disagree
- Disagree
- Neutral
- Agree
- Strongly Agree

23. I did not need more references in the explanations: e.g.,medical guidelines, regulations to understand the web-based prognostic tool. *

Mark only one oval.

- Strongly disagree
- Disagree
- Neutral
- Agree
- Strongly Agree

24. I received the explanations and prediction of locoregional recurrence in a timely and efficient manner. *

Mark only one oval.

- Strongly disagree
- Disagree
- Neutral
- Agree
- Strongly Agree

Part III: Attitude towards Artificial Intelligence in cancer managment.

Artificial Intelliegence and its subfields such as machine learning, natural lanaguage processing etc

25. The use of artificial intelligence (and its subfields) will contribute effectively to proper cancer management in the future. *

Mark only one oval.

- Strongly disagree
- Disagree
- Neutral
- Agree
- Strongly Agree

General comments

Do you have any comment for us? You are welcome to do so.

26. Any comments?

____

THANK YOU.

We appreciate your time to take part in the survey.

For comments/suggestions, contacts.......

Alabi Rasheed Omobolaji
Postdoctoral Researcher
Biomedicum - Research Program in Systems Oncology
Faculty of Medicine, University of Helsinki, Helsinki, Finland.
rasheed.alabi@helsinki.fi

This content is neither created nor endorsed by Google.

Google Forms
